# Cholesterol Interaction with the MAGUK Protein Family Member, MPP1, via CRAC and CRAC-Like Motifs: An In Silico Docking Analysis

**DOI:** 10.1371/journal.pone.0133141

**Published:** 2015-07-17

**Authors:** Marcin A. Listowski, Jacek Leluk, Sebastian Kraszewski, Aleksander F. Sikorski

**Affiliations:** 1 Department of Cytobiochemistry, Faculty of Biotechnology, University of Wrocław, Wrocław, Poland; 2 Department of Molecular Biology, University of Zielona Góra, Zielona Góra, Poland; 3 Department of Biomedical Engineering, Wrocław University of Technology, Wrocław, Poland; College of Medicine, University of South Florida, UNITED STATES

## Abstract

Cholesterol is essential for the proper organization of the biological membrane. Therefore, predicting which proteins can bind cholesterol is important in understanding how proteins participate in lateral membrane organization. In this study, a simple bioinformatics approach was used to establish whether MPP1, a member of the MAGUK protein family, is capable of binding cholesterol. Modelled and experimentally-validated fragment structures were mined from online resources and searched for CRAC and CRAC-like motifs. Several of these motifs were found in the primary structure of MPP1, and these were structurally visualized to see whether they localized to the protein surface. Since all of the CRAC and CRAC-like motifs were found at the surface of MPP1 domains, in silico docking experiments were performed to assess the possibility of interaction between CRAC motifs and cholesterol. The results obtained show that MPP1 can bind cholesterol via CRAC and CRAC-like motifs with moderate to high affinity (K_I_ in the nano- to micro-molar range). It was also found that palmitoylation-mimicking mutations (C/F or C/M) did not affect the affinity of MPP1 towards cholesterol. Data presented here may help to understand at least one of the molecular mechanisms via which MPP1 affects lateral organization of the membrane.

## Introduction

A specific sequence motif, termed the cholesterol recognition/interaction amino acid consensus (CRAC) [1], is present in many proteins that are known to interact with cholesterol. It consists of branched apolar amino-acid residues, L and V, followed by any residues in a 1–5 segment, then a mandatory aromatic residue Y and, then again, a segment of 1–5 of any residues, and ends with K or R. Looseness of the motif definition caused some scepticism about its predictive value [2–3]. However, this motif is present in many proteins that are known to bind cholesterol and, in many cases, the interaction was confirmed by physicochemical approaches.

Occurrence of the CRAC motif within the transmembrane segment of a protein increases the reliability of prediction of cholesterol interaction with such a protein. The CRAC motif can also be present in proteins that are not integrated with a biomembrane. A good example of this is cytolethal-, distending toxin C of *Aggregatibacter actinomycetemcomitans* (CdtC). This protein contains a CRAC sequence motif, LIDYKGK, which is exposed at the surface of the protein and binds to liposomes containing cholesterol. Mutation of the Y residue to P results in reduced binding to cholesterol-containing membranes and to target cells, which reduces its toxicity [4]. CRAC motifs have also been found on the surface of viral proteins. HIV-1 fusion protein gp41 contains a CRAC motif, which is localized to the extracellular surface of the membrane, adjacent to the transmembrane segment of this protein. It has been suggested that this protein is important for membrane fusion [5–6]. Depletion of cholesterol from infected cell membranes reduces virus release, and released HIV virions show minimal infectivity [7]. HIV-1 is known to enter the cell through binding to the CD4 receptor [8], which is associated with a cholesterol-rich raft region [9–10]. This raises the possibility that the CRAC motif in the gp41 protein interacts with the cholesterol in the host cell-membrane. Protein interaction with cholesterol via the CRAC motif was also demonstrated for human type-1 cannabinoid receptor (CB_1_R). Point mutation in the CRAC lysine residue K402G resulted in the receptor becoming insensitive to membrane cholesterol [11].

Apart from CRAC, another motif with a very similar sequence, termed CARC or CRAC-like motif, has been proposed [12]. It is sometimes referred to as an “inverted CRAC”. Reverse orientation is not the only feature which distinguishes it from CRAC. Another difference is that a central residue can be either Y or F. Docking studies demonstrated a favorable fit of cholesterol to both Y and F containing motifs [12]. Also, in biochemical studies, substitution of the Y residue with an F did not abolish the redistribution of cholesterol [13].

Membrane Palmitoylated Protein 1 (MPP1) is a member of the membrane-associated guanylate kinase homologues (MAGUK) protein family. Proteins of this family share similar domains, such as a PDZ domain, SH3 (src-homology-3) motif and a GUK domain (domain homologous to guanylate kinases) [14–16]. It was originally identified as a membrane skeletal protein in erythrocytes, where it stabilizes the spectrin-actin skeleton by interacting with glycophorin C and protein 4.1R [17–19]. MPP1 is a major target of palmitoylation in red blood cells. Lack of protein acyltransferase (PAT) activity in these cells causes changes in lateral membrane organization, which is, as we suggest, an impairment of resting-state raft formation. This was revealed by several methods [20]. Moreover, silencing of *MPP1* gene expression in human erythroleukemia (HEL) cells results in similar changes in membrane lateral organization [21]. Since cholesterol is a major component of raft domains, the mechanism of their formation could include interaction between MPP1 protein and cholesterol. However, no interaction of cholesterol with human MPP1 protein has been demonstrated to date.

Here we present an in silico analysis of cholesterol interaction with MPP1 protein. The results obtained show that there is a possibility of such an interaction. These results provide a new insight on at least one possible molecular mechanism of resting-state raft formation and function of MPP1.

## Materials and Methods

### Identification of CRAC and CRAC-like motifs

The sequence of MPP1 was obtained in FASTA format from UniProt (http://www.uniprot.org/). A search for CRAC and CRAC-like motifs was then performed with EMBROSS: fuzzpro (http://emboss.bioinformatics.nl/cgi-bin/emboss/fuzzpro). Sequences given as a search pattern were: [LV]-X(1,5)-Y-X(1,5)-[RK], [RK]-X(1,5)-Y-X(1,5)-[LV], [LV]-X(1,5)-F-X(1,5)-[RK], [RK]-X(1,5)-F-X(1,5)-[LV] [1,12].

### Experimental structures

For searching existing experimental structures (obtained either by NMR or X-Ray diffraction), The Protein Model Portal (http://www.proteinmodelportal.org/) was used. The complete sequence of human MPP1 protein was entered as a query. Three experimentally validated partial-structures of MPP1 protein were obtained and downloaded from Protein Data Bank (http://www.rcsb.org), Namely, the PDZ domain [2ev8] [22], the PDZ domain complexed with glycophorin C [2ejy] [23] and the GUK domain [3ney] were used. The sequence range of these results were then compared with CRAC and CRAC-like motifs in the primary structure of the MPP1 protein. If the sequence range of experimental structures contained cholesterol-binding motifs, these structures were chosen for further processing.

### Building the full model structure

Since there was no single complete protein structure of MPP1 published, to build a full model structure, CASP7, 8, 9 and 10 winning web server, I-TASSER was chosen to assemble a complete structural model of MPP1 [24–26]. The full amino-acid sequence of MPP1, obtained from Uniprot (http://www.uniprot.org/), was given as a query. The web server operated at its default setting, that is, no specification of templates was given. Also, no assignment of contact or distance restraints was given. The server was also set to not exclude any homologous or specific templates. In brief, the templates selected by the server corresponded to the three main domains of MPP1, ie. PDZ, SH3 and GUK, as well as the inter-domain regions. Since the full structure of MPP1 remains unknown, the top template which was selected by the server was one for another MAGUK family member, PSD-95, whose structure has been fully resolved. To validate the correctness of the server selection, known fragments of MPP1 (PDZ and GUK domains) were compared to those of PSD-95 by the TM-align online tool [27] and were found to be structurally similar (TM-score 0,72 and 0,76 respectively). Structural similarity was also good for the constructed model when compared with known fragments of MPP1 (TM-score 0,74 for PDZ domain and 0,77 for GUK domain). The top-ranked model constructed by the server was used in further in silico experiments.

### Visualisation of CRAC motifs

Accelrys Discovery Studio v3.5.0.12158 was used for visualising selected motifs in both the experimental and model structures. If the experimental structure was chosen, identified motifs were visualised only on a single chain of a protein.

### Hydrophobicity estimation

The primary structure of MPP1 was used for hydrophobicity estimation. Two separate scales, namely the Wimley-White [28] and Eisenberg [29] scales, were used to deal with this task. A profile for the Wimley-White scale was generated using the Mpex v. 3.2.6. (19 AUG 2013) program (http://blanco.biomol.uci.edu/mpex/). Partitioning was set to “Water to Bilayer”. D and E residues were set as charged. H residues were set as neutral. Along with a hydrophobicity plot, hydrophobic-moment plot and text results were also generated. All other settings were left at default values. Since salt-bridges can increase the affinity of a protein towards the cell-membrane [30], and that Mpex v. 3.2.6. is able to take this factor into account, the ESBRI web server [31] was used to predict possible salt-bridges in the full model of MPP1. The profile results for the Eisenberg scale were generated using an online ProtScale tool [32] (http://web.expasy.org/protscale/). Window size was set to 9 and the weight variation model was set to linear. No scale normalization was performed.

### Docking experiments

Docking experiments were performed using AutoDock v4.2.5.1 with AutoDockTools version 1.5.6rc3. The Cholesterol *.pdb file used for the experiments was obtained from http://cat.middlebury.edu/~chem/chemistry/pdb/cholesterol.pdb. Appropriate experimental structures, or a model structure were loaded into the program. In the next step, the grid box was created with a density of at least 0,35 Å. The grid box was set to fully cover the CRAC or CRAC-like sequence motifs and some additional residues overlapping them. Automated docking using the Lamarckian Genetic Algorithm was performed to search for best fit of cholesterol and for estimating the score binding energy and K_I_ values. Gasteiger charges were applied to both receptor and the ligand. The receptor (either the model or the experimental structure of MPP1) was treated as a rigid body, while the ligand (cholesterol) was treated as being flexible. Six torsions were set for the ligand. Docking was performed with a maximum number of energy evaluations of 2,500,000, the number of individuals in the population of 300 and the number of algorithm runs set to 100. Other parameters were left at their default values. Results were visualised in AutoDockTools version 1.5.6rc3. Along with visualisation, the binding energy and K_I_ values were determined for each predicted binding site.

## Results

### Cholesterol-binding motifs are present in the primary structure of MPP1

The CRAC motif is defined by the pattern–L/V-X_1–5_-Y-X_1–5_-R/K–. In our analyses, using EMBROSS: fuzzpro (http://emboss.bioinformatics.nl/cgi-bin/emboss/fuzzpro), we searched the primary structure of MPP1 for this pattern as well as for its reverse form,–R/K-X_1–5_-Y-X_1–5_-L/V–(CARC or CRAC-like motif), along with motifs that possesses a central phenylalanine residue instead of tyrosine (CRAC-like). Using these criteria, we found 21 motifs in the complete sequence of the human MPP1 protein, grouped in six larger sequence-fragments. They either overlap each other or occur individually, without having common residues. In the sequence range between residues 258 and 286, a total of eight motifs, both overlapping and individually occurring, were identified. The sequence fragments identified, together with their residue range and total number of identified motifs, both overlapping and individually occurring, are presented in Table 1.

**Table 1:** Sequence fragments containing cholesterol-binding motifs in the primary structure of MPP1. Almost all fragments (except one, residues 423–434) contain more than one possible cholesterol-binding motif, with sequence fragment 258–286 having a total of eight of them. These motifs either overlap each other or occur individually.

Sequence motif	Sequence range	Total motifs in sequence
KVRLIQFEKV	68–77	4
LPALQMFMR	156–164	2
KKKKYKDKYL	247–256	3
KHSSIFDQLDVVSYEEVVRLPAFKRKTLV	258–286	8
KFVYPVPYTTRPPR	309–322	3
RSQYAHYFDLSLV	423–435	1

### Cholesterol-binding motifs are located at the surface of the MPP1 protein

**Fig 1:**
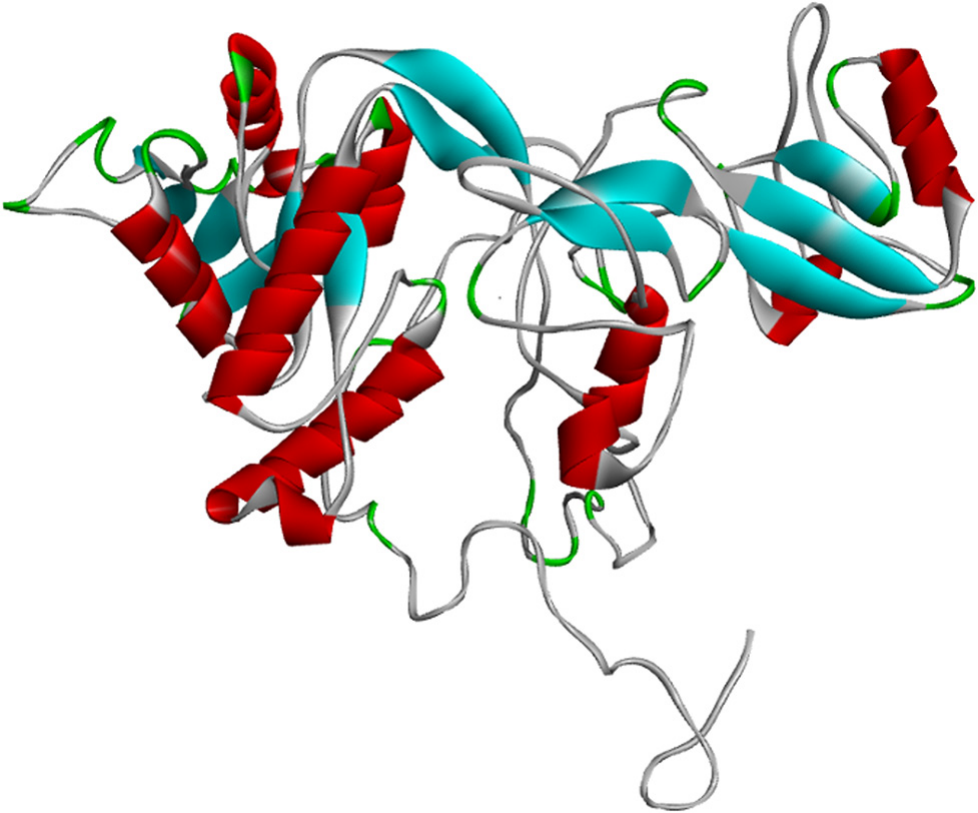
Three dimentional structure of MPP1 built with the I-TASSER web server. Alpha-helical structures are presented in red. Beta-strand structures are presented in blue. Coils are indicated by green colouring and unstructured regions are in grey.

**Fig 2:**
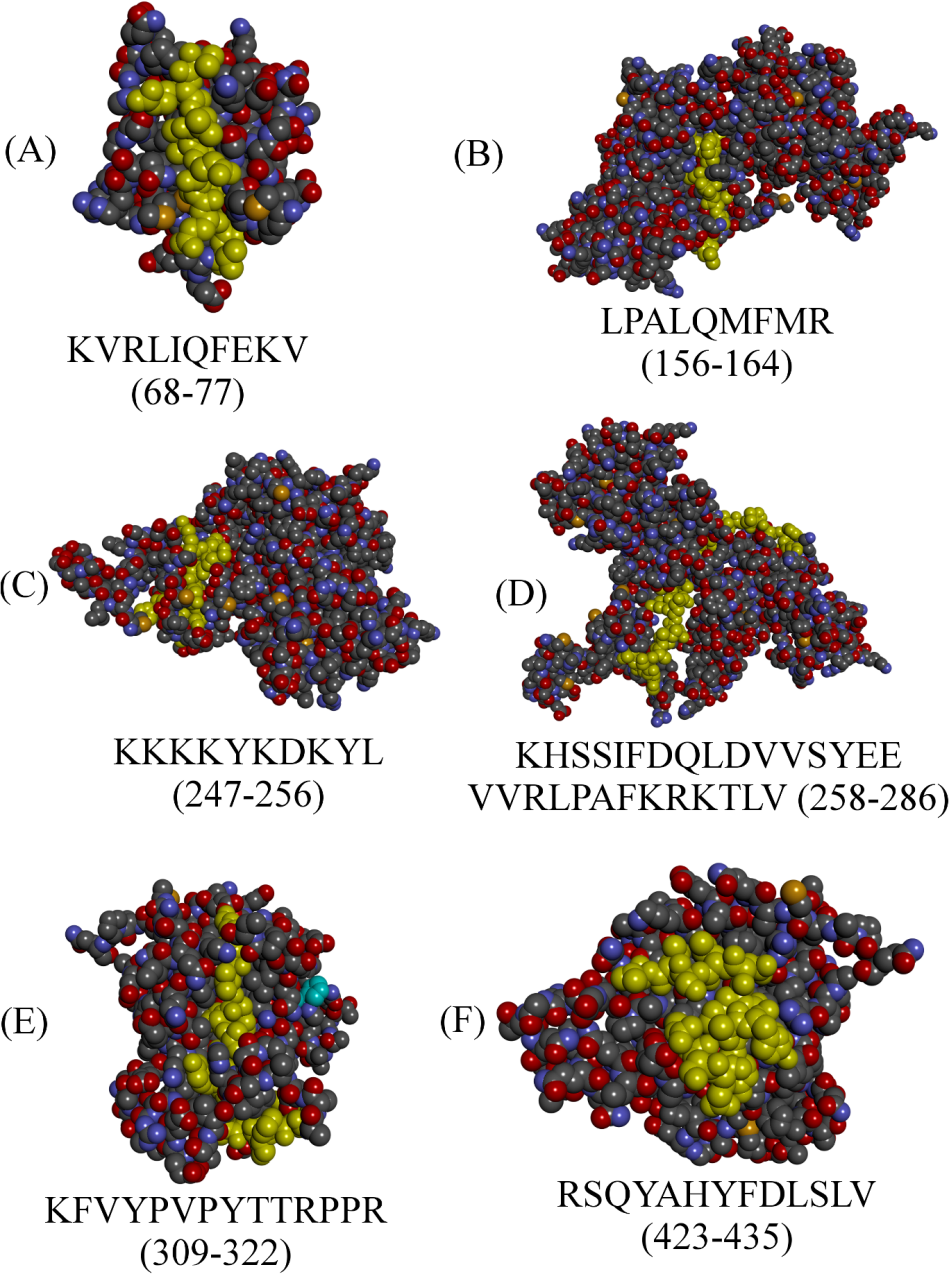
Visualization of possible cholesterol-binding motifs in the human MPP1 protein. Motifs in B, C and D are presented on the full model structure. A, E and F are presented on experimentally obtained structures. All residues are presented in CPK display and those corresponding to CRAC, CRAC-like and CARC motifs are highlighted in yellow. Visualized with Accelrys Discovery Studio v3.5.0.1215.

The complete, experimentally validated structure of MPP1 is not available. Therefore, a full model of the protein was built on the I-TASSER web server (Fig 1). When possible, motifs were first visualised on experimentally-obtained structures. However, this was only possible for three of them (residue ranges 68–77, 309–322 and 423–435). Since online databases host three partial-model structures, the full-model option was chosen for visualisation purposes. This is due to the fact that partial models may not allow for prediction of whether residues belonging to CRAC and CRAC-like motifs are not covered by residues coming from outside of those included in the partial model. The same situation also applies to experimentally-obtained partial structures. However, taking into account that experimental structures are much more accurate, visualisation was performed using actual structure information wherever possible, rather than model data. All cholesterol-binding motifs that were identified in various parts of the protein were found to be exposed to the surface of the protein (Fig 2).

### MPP1 contains hydrophobic regions

**Fig 3:**
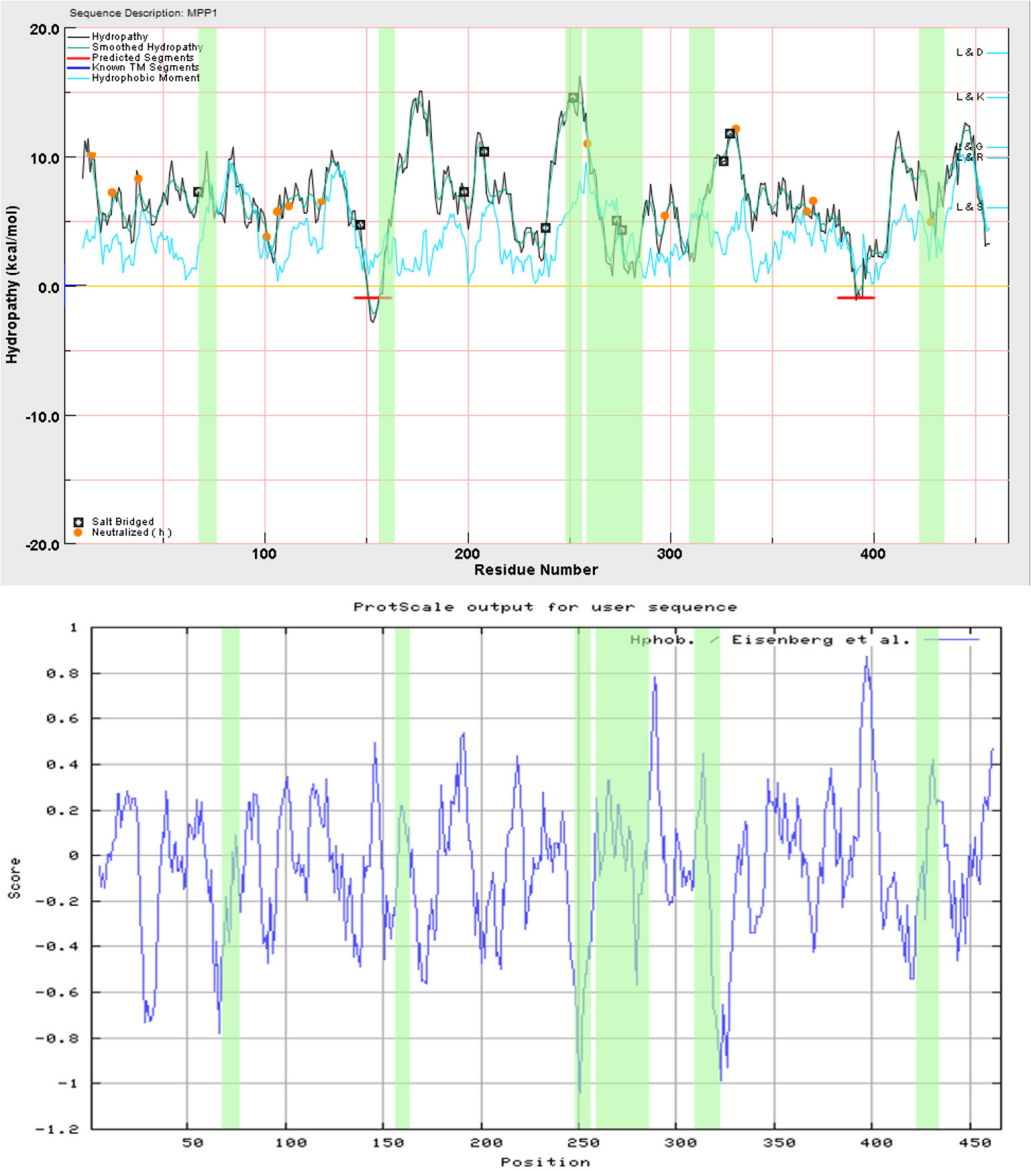
Hydrophobicity profiles of MPP1 plotted using the Wimley-White scale (top) and the Eisenberg scale (bottom). The profile for the Wimley-White scale was plotted using Mpex v. 3.2.6. The profile for the Eisenberg scale was plotted using ProtScale. Green areas correspond to identified cholesterol-binding motifs. Dark squares represent residues involved in the formation of salt-bridges. The light-blue graph corresponds to hydrophobic moment. Orange dots represent all of the histidine residues in the MPP1 sequence, which were assumed as uncharged. According to the Wimley-White scale, sequence fragment LPALQMFMR (156–164), apart from its last two residues, is located in a possible membrane-penetration region (indicated by the red line).

Since cholesterol is located mostly in the hydrophobic core of the cell membrane, protein interaction with it would require at least partial membrane-penetration by hydrophobic residues of the protein. Such a situation is documented for Caveolin-1, where the CRAC motif is followed by a membrane penetrating region, facilitating membrane-domain formation in DPPC and cholesterol-containing vesicles [33]. To check the hydrophobicity of MPP1, the Wimley-White and Eisenberg scales were used. The ESBRI web server predicted six possible salt-bridges in the MPP1 structure. Five of them were included in the hydrophobicity estimation. The sixth salt-bridge included histidine 428. Since histidine residues were assumed as uncharged, it was not included in the hydrophobicity estimation. A very strong hydrophobic region was identified by both scales. It includes residues from 382 to 400 (Wimley-White scale) and 391 to 403 (Eisenberg scale). The Wimley-White scale also disclosed one strong hydrophobic region between residues 144 and 162. What is interesting is that this region resembles closely that of caveolin-1, which is involved in interaction with cholesterol [33]. Both regions differ from each other, however, by their hydrophobic moments. The low energy-transfer value for the region comprising residues 382 to 400 of MPP1 is consistent with low hydrophobic moment. On the other hand, for the region comprising residues 144 to 162, the low energy transfer value contrasts with a higher hydrophobic moment. The Eisenberg scale, in contrast to the Wimley-White scale, also predicts a strong hydrophobic region between residues 287 and 292. The global maximum of hydrophobicity was found around residue 390. Profiles using both scales are presented in Fig 3.

### Cholesterol binds to located sequence motifs in silico

**Fig 4:**
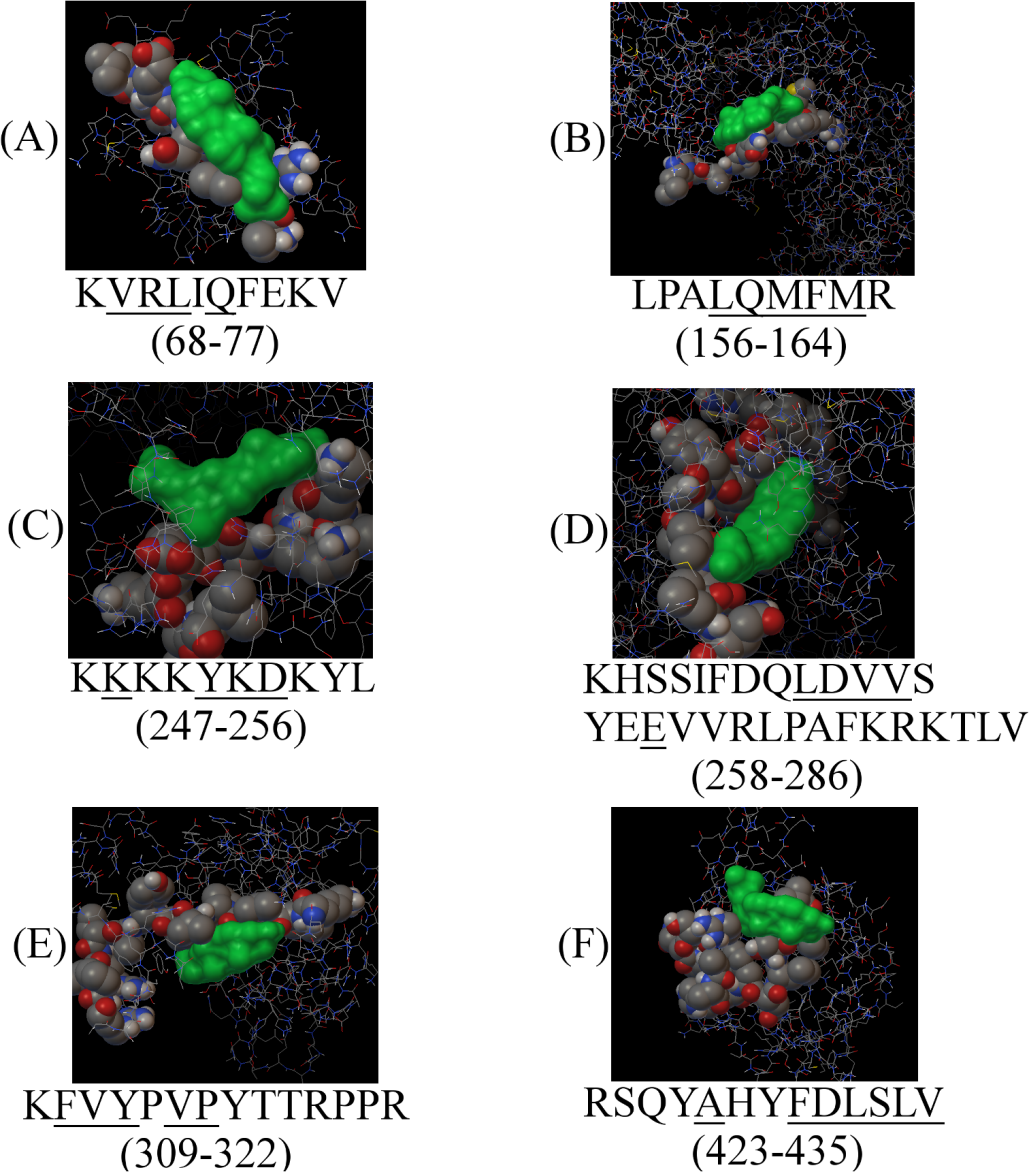
Graphi-cal output of docking experiments to cholesterol-binding motifs in the primary structure of the MPP1 protein. Cholesterol is presented in monochrome green, binding motifs are presented in the CPK display. Underlined are amino-acid residues involved in cholesterol-binding. Obtained with AutoDock v4.2.5.1 and AutoDockTools version 1.5.6rc3.

Using the full model and experimentally-defined structures, *in silico* docking experiments were preformed to test whether cholesterol-binding to these motifs was possible. Docking results show that cholesterol binds to these sequence motifs with a binding-energy ranging from -6.23 to -10.52 kcal/mol and K_I_ values ranging from 0.019 to 27.12 μM. Docking results for all tested motifs and a comparison of the results obtained from both experimental and model structures are presented and summarized in Table 2. Fig 4 contains graphical representations of these data.

**Table 2:** *In silico* data obtained for cholesterol docking to CRAC and CRAC-like motifs in human MPP1. Data obtained by using AutoDock v4.2.5.1 and AutoDockTools version 1.5.6rc3.

Sequence motif	Experimental		Model	
	K_I_ [μM]	Binding energy[kcal/mol]	K_I_ [μM]	Binding energy[kcal/mol]
KVRLIQFEKV (68–77)	27.12	-6.23	3.61	-7.43
LPALQMFMR (156–164)	——	——	0.081	-9.67
KKKKYKDKYL (247–256)	——	——	0.067	-9.78
KHSSIFDQLDVVSYEEVVRLPAFKRKTLV (258–286)	——	—-	0.309	-8.88
KFVYPVPYTTRPPR (309–322)	0.518	-8.57	0.019	-10.52
RSQYAHYFDLSLV (423–435)	23.91	-6.30	0.561	-8.53

#### KVRLIQFEKV (68–77)

This sequence range contains four overlapping putative binding motifs. Seven residues from the C terminus locate to the PDZ domain of MPP1. This suggests the possibility of interaction with cholesterol, and this domain has been shown previously to bind cholesterol [34]. However, the predicted K_I_ value is relatively high for the docking preformed on the experimental structure (Table 2), and the sequence itself corresponds to a rather hydrophilic region of the protein. This greatly reduces the possibility of cholesterol binding.

#### LPALQMFMR (156–164)

According to the Wimley-White scale, sequence fragment LPALQMFMR (amino acid residues potentially interacting with cholesterol are underlined), which is located within the SH3 domain of MPP1, is situated in a very hydrophobic region of the protein. The sequence fragment itself contains two overlapping CRAC-like motifs. Interacting amino-acid residues correspond to the shorter of the two motifs, which begins with the second leucine residue from the N-terminus. Both motifs include the common F residue substituted for Y. This kind of substitution does not abolish interaction with cholesterol [13] and our docking studies show rather a high affinity of cholesterol towards this motif (Table 2). This means that cholesterol interaction with the CRAC-like motif occurring within the LPALQMFMR sequence fragment is highly possible.

#### KKKKYKDKYL (247–256)

The sequence fragment characterized by low binding-energy and the lowest K_I_ value is fragment KKKKYKDKYL. On the other hand, it is composed mostly of lysine residues, making it very hydrophilic, as indicated by both hydrophobicity scales. Therefore, it is rather unlikely that this sequence fragment would be embedded in the cell membrane.

#### KHSSIFDQLDVVSYEEVVRLPAFKRKTLV (258–286)

Fragment KHSSIFDQLDVVSYEEVVRLPAFKRKTLV contains the largest number of cholesterol-binding motifs. A total of eight (two individual and two overlapping triplets) such motifs can be identified in the given sequence. Underlined, are the residues responsible for interacting with cholesterol KHSSIFDQLDVVSYEEVVRLPAFKRKTLV with parameters given in Table 2. These residues belong to the CRAC motif, **L**DVVS**Y**EEVV**R**. Moreover, the calculated binding-energy and the K_I_ value are in the range of high possibility of interaction. According to the Eisenberg scale, this motif is located within a weak hydrophobic region, neighbouring with a region of increased hydrophobicity.

#### KFVYPVPYTTRPPR (309–322)

The KFVYPVPYTTRPPR sequence fragment is composed of one CARC, **K**FV**Y**P**V,** and two overlapping CRAC motifs, **V**P**Y**TTRPP**R** and **V**YPVP**Y**TTRPP**R**. Docking analyses showed that residues involved in cholesterol interaction belong to all of these motifs. Also, the binding energy and K_I_ value are low, at -8.57 kcal/mol and 518 nM, respectively, for the experimentally obtained structure, and even lover for the full protein model (Table 2). This sequence-fragment is located in the guanylate kinase-like domain of MPP1. Due to its location in a hydrophobic region, and its binding parameters, this motif could also be involved in interaction with cholesterol.

#### PQTLKIVRTAELSPFIVFI (382–400) and RSQYAHYFDLSLV (423–435)

According to the Eisenberg scale, residues in sequence-fragment RSQYAHYFDLSLV are located in the hydrophobic region of the protein. However, docking experiments performed on experimentally-obtained structure data indicate a relatively high binding-energy and corresponding K_I_ value between this sequence and cholesterol. This fact makes interaction with cholesterol less likely. In our docking analyses we also tested the most hydrophobic region of the protein, namely PQTLKIVRTAELSPFIVFI, located between residues 382–400 (Fig 3). Whilst this region does not contain any cholesterol interacting elements, since it is located in the most hydrophobic region of the molecule, it was interesting to see its interaction parameters with cholesterol. Binding energy and K_I_ values were -8.8 kcal/mol and 357.28 nM respectively.

Since three docking experiments were performed on experimental structures using motifs located at residue ranges of 68–77, 309–322 and 423–435, it was also interesting to see whether docking to a model structure would give similar results. Therefore, docking experiments were repeated with the parameters given in Materials and Methods. Comparison of docking results to model and experimental structures are presented in Table 2.

Docking to the model structure results in lower binding-energies and more negative K_I_ values, in comparison to the same experiments performed on experimentally obtained structures (Table 2).

Furthermore, we tested whether protein palmitoylation affects docking of cholesterol. To simulate such a situation, we substituted cysteine 242, which was found at a physiological palmitoylation site [35] on MPP1, with phenylalanine [36] or with methionine. Palmitoylation has been shown to be crucial in localization of proteins to DRMs, as its inhibition by 2-bromopalmitate removes proteins from DRMs, as shown by our previous studies [21]. Substitution of cysteine by a phenylalanine residue does not abolish the ability of the protein to localize to DRMs and, therefore, can serve as an experimental mimic of palmitoylation [37]. Docking was then performed for all fragments containing possible cholesterol-binding motifs. It was impossible to test the effects of mutation on experimental structures, since none of them contained the cysteine residue of interest. Mutation effects were therefore examined on the model structure in which cysteine was substituted with phenylalanine or methionine. Docking experiments were performed with the same grid-settings as for the wild-type model. We found that both substitutions did not have a significant effect on cholesterol-binding. Depending on the motif, binding affinity, as determined by the binding-energy and K_I_ value, was either slightly increased or decreased (S1 Table). This suggests that substitution of cysteine 242 and furthermore, its palmitoylation, does not have a significant effect on the affinity of MPP1 towards cholesterol.

It was also checked whether palmitoylation-mimicking mutations would have the same effect on H-Ras, as was found for MPP1. Palmitoylation of H-Ras occurs on residues C181 and C184 [38]. However, these residues are not in the range of any experimentally-defined structures. It was then decided to build a full model-structure of H-Ras using the same approach as that used for MPP1. Models containing palmitoylation-mimicking mutations in both cysteine residues were also built. It was interesting to find that the results from this analysis differ from those obtained for MPP1. Docking to the experimental [39] and model structures of H-Ras produced similar results in terms of binding energies and affinities, determined at -7.97 kcal/mol and K_I_ of 1.45 μM vs -7.3 kcal/mol and K_I_ of 4.48 μM, respectively. However, the binding-affinity was slightly higher for the experimental structure than for the model. In addition, docking experiments on palmitoylation-mimicking mutants showed that simultaneous substitutions of both cysteine residues with phenylalanine increases the affinity of H-Ras towards cholesterol (-9.57 kcal/mol vs -7.3 kcal/mol), while methionine substitutions had little effect (-7.63 kcal/mol vs -7.3 kcal/mol).

**Fig 5:**
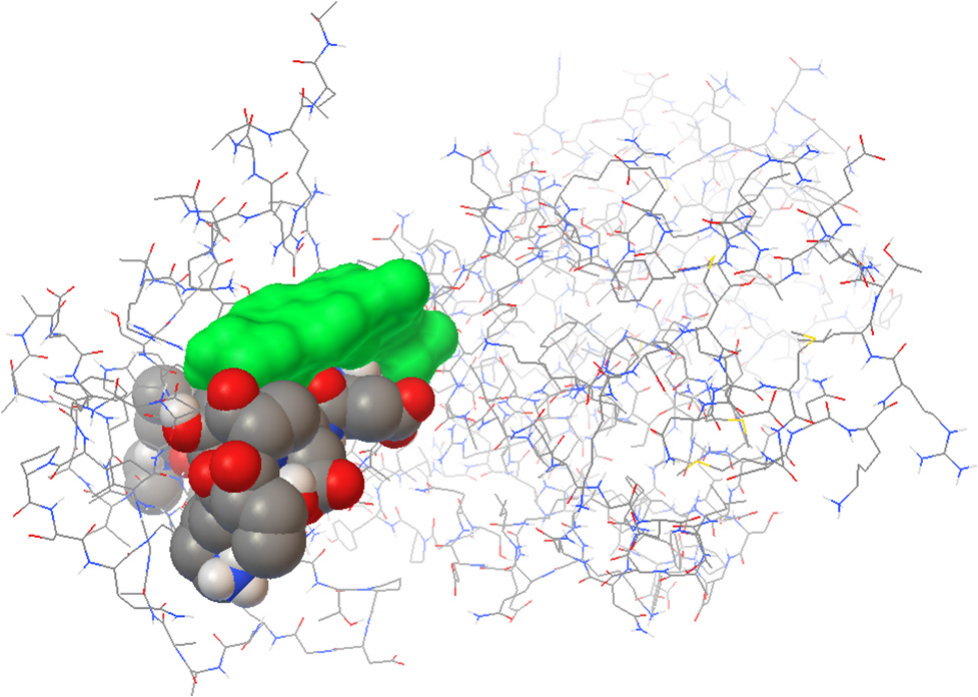
Cholesterol (monochrome green) docked to the SH3 domain of the DLG1 protein (CPK display), containing a CRAC motif. For details see Fig 2 legend.

As MPP1 belongs to the large MAGUK protein family, it was interesting to see whether other proteins within the family have similar motifs. As shown in Fig 5, DLG1 protein shows at least one surface-exposed cholesterol-binding site in its SH3 domain. Docking results indicate a moderate affinity for this binding, with an energy of -7.99 kcal/mol and K_I_ of 1.39 μM.

In conclusion, the data presented above indicate a possibility of the existence of surface-located cholesterol-binding sites in MPP1, which is a known peripheral membrane protein. Moreover, the results of docking experiments presented implicate the involvement of the cholesterol-binding properties of MPP1 protein in the interaction with membrane cholesterol and therefore with its complexes with membrane lipids and proteins, which could explain MPP1 involvement in resting-state membrane-raft formation.

## Discussion

Our simple modelling study showed the existence of multiple, surface-located, cholesterol-binding consensus motifs in the MPP1 molecule. Moreover, theoretical docking analyses confirmed the presence of moderate-to-high affinity binding-site properties for cholesterol for these consensus sequence motifs. However, care is needed in assigning function based on modelling. By way of example, studies performed on the nicotinic acetylcholine receptor (nAChR) highlight the problem of variability in such predictions. However, the docking studies conducted on nAChR were further validated by MD simulations, where it was found that complexes of cholesterol with nAChR were stable under these simulations [40]. These simulations were strengthened by experimental data which showed that nAChR indeed binds cholesterol at multiple sites [41]. Another example is the TRPV1 ion channel. This protein has a CRAC motif which binds cholesterol in in silico simulations. The interactions were further confirmed by point mutation in the motif, which abolished the cholesterol inhibition effect on the TRPV1 ion channel [42]. While the data obtained for MPP1 needs to be confirmed experimentally, according to our best knowledge, this is the first indication of such an activity for this protein. Since the criteria for consensus sequences are not as strict as they are for docking, two series of comparisons were performed. First, we searched several SH3 domains from various proteins in order to see whether a cholesterol-binding sequence motif could be detected and, second, a similar analysis involving both surface localization and docking experiments was performed on the H-Ras protein, which is known to be raft-associated [43]. As can be seen from the data presented in Table 3, only two of all of the examined SH3 domains do not contain CRAC or CRAC-like binding motifs.

**Table 3:** SH3 domains of various MAGUK proteins. Possible cholesterol-binding motifs identified are shown in bold. Some of these motifs group together, forming larger conglomerates which contain more than one motif (similar to MPP1). The SH3 domains of two of these proteins, CASK and ZO2, do not contain any cholesterol-binding motifs.

Protein	SH3 domain
MPP1	A**LQMFMR**AQFDYDPKKDNLIPCKEAGLKFATGDIIQIINKDDSNWWQGRVEGSSKESAGLIPSPELQEWRV
MPP2	PRQ**VFVKCHFDYDPAR**DSLIPC**KEAGLRFNAGDLL**QIVNQDDANWWQACHVEGGSAGLIPSQLLEEKRK
MPP3	ES**KVFMRALFHYNPR**EDRAIPCQEAG**LPFQRR**QVLEVVSQDDPTWWQAKRVGDTNLRAG**LIPSKGFQERRL**
MPP4	QQMVYVRAMTEYWPQEDPDIPCMDAG**LPFQK**GDILQIVDQNDALWWQARKISDPATCAGLVPSNHLLKRKQ
MPP5	ETVIHVKAHFDYDPSDDPYVPC**RELGLSFQKGDIL**HVISQEDPNWWQAYREGDEDNQPLAG**LVPGKSFQQQR**E
MPP6	PQQVFVKCHFDYNPYNDNLIPC**KEAGLKFSKGEIL**QIVNREDPNWWQASHVKEGGSAG**LIPSQFLEEKRK**
MPP7	EG**KMFIKALFDYNPNEDK**AIPC**KEAGLSFKKGDIL**QIMSQDDATWWQAKHEADANPRAG**LIPSKHFQERRL**
DLG1	**KRSLYVRALFDYDKTK**DSGLPSQGLNFKFGDILHVINASDDEWWQARQVTPDGESDEVGVIPSKRRVEKKE
DLG2	P**VAFAVR**TNVGYNPSPGDEVPVQG**VAITFEPK**DFLHIKEKYNNDWWIGRLVKEGCEVGFIPS
DLG3	S**LYVRALFDYDRTR**DSCLPSQGLSFSYGDILHVINASDDEWWQARLVTPHGESEQIGVIPSKKRVE
DLG4	**KRGFYIRALFDYDKTKDCGFLSQAL**SFRFGDVLHVIDASDEEWWQARRVHSDSETDDIGFIPSKRRVERRE
DLG5	GDSFYI**RALYDRLADVEQELSFKKDDILYVDDTL**PQGTFGSWMAWQLDENAQKIQRGQIPSKYVMDQEF
ZO1	GDSFYIRTHFEYE**KESPYGLSFNKGEVFRVVDTLYNGKL**GSWLAIRIGKNHKEVERGIIPNKNRAEQLA
ZO2	GDSFFIRSHFECEKETPQSLAFTRGEVFRVVDTLYDGKLGNWLAVRIGNELEKGLIPNKSRAEQMA
ZO3	GDSFYI**RTHFEL**EPSPPSG**LGFTRGDVFHVLDTL**HPGPGQSHARGGHWLAVRMGRDLREQERGIIPNQSRAEQLA
CASK	IYVRAQFEYDPAKDDLIPCKEAGIRFRVGDIIQIISKDDHNWWQGKLENSKNGTAGLIPSPELQEWRV

Analysis of the experimentally-obtained structure of H-Ras [39] showed the presence of a single, surface-exposed CRAC motif, and docking experiments characterize it with moderate binding affinity (energy of -7.97 kcal/mol and K_I_ of 1.45 μM). In comparison, the cholesterol-binding sites identified in the MPP1 molecule show a higher affinity towards cholesterol, with the highest affinities obtained for the motif located within residues 309–322 of MPP1, displaying a K_I_ of 19 nM for the model structure and a K_I_ of 518 nM for the experimentally-obtained structure (Table 2). In contrast, a palmitoylation-mimicking mutation (substitution of cysteine with phenylalanine) increased the affinity of H-Ras towards cholesterol, resulting in a K_I_ of 1.45 μM, compared to a K_I_ of 96.6 nM for the non-palmitoylated form. Therefore, the affinity of palmitoylated H-Ras is still lower than that for the model structure of MPP1, having a K_I_ of 96.6 nM as opposed to the K_I_ of 19 nM for the MPP1 model, but it is higher than that for the MPP1 experimental structure, having a K_I_ of 96.6 nM versus 518 nM. The fact that H-Ras localizes to cholesterol-rich membrane domains [43–44], as well as the fact that the obtained values of K_I_ for both H-Ras and MPP1 are not substantially different, provides support for the notion that MPP1 could interact with membrane-cholesterol. This fact, in turn, could explain one of the possible mechanisms of resting-state raft formation.

The presence of multiple cholesterol-binding sites in MPP1 could explain at least one of the possibilities of its involvement in resting-state membrane-raft formation. As was shown in our previous work [20–21], the lack of the palmitoylated form of protein MPP1 in erythroid cells induced dramatic change in lateral membrane organization, i.e. impairment of “resting-state raft” formation, which was manifested as a decreased DRM fraction and decreased order of the membrane in FLIM experiments. It was hypothesised that MPP1 is involved in oligomerization of unstable (<0.1 ms), pre-existing, nanocluster complexes (<10 nm in diameter) of membrane-raft lipids and proteins into functional “resting-state rafts”, which are larger (~20 nm) in diameter [45], more stable (10–20 ms), functional and detergent-resistant domains. The results presented here, indicating the presence of multiple cholesterol-binding sites of moderate-to-high affinity, could provide a mechanism for MPP1 action in organizing membrane-rafts, based upon its interaction with cholesterol. Multivalent protein (possessing more than one cholesterol-binding site) would be responsible for the oligomerization of pre-existing nanoclusters, leading to the resting-state raft formation.

Although this hypothesis seems plausible, it requires experimental confirmation. Further, it does not exclude other possibilities, which include the involvement of different membrane-raft lipids, such as phosphatidyl inositol derivatives, or other proteins, such as flotillin 1 or 2 binding. It could also be the case that one of the MPP1 cholesterol-binding sites is occupied by cholesterol, while the other is occupied by PIP2, or flotillins.

In conclusion, we have shown the possibility of multiple cholesterol-binding motifs in the MPP1 molecule. Docking experiments confirmed the possibility of cholesterol-binding. This binding could be considered at least as one of the possible mechanisms of resting-state membrane raft formation.

## Supporting Information

S1 Table Comparison of docking results for palmitoylation mimicking mutations in MPP1. (DOC)
