# Supplementary material for: Cholesterol Interaction with the MAGUK Protein Family Member, MPP1, via CRAC and CRAC-Like Motifs: An In Silico Docking Analysis
Source: PLoS One. 2015 Jul 17;10(7):e0133141. doi: 10.1371/journal.pone.0133141 (PMC4505867; doi:10.1371/journal.pone.0133141)
Supplement: S1 Table — (DOC) [file pone.0133141.s001.doc]

S1 Table. Comparison of docking results for palmitoylation mimicking mutations in MPP1.

| Sequence motif | WT | | Phenylalanine | | Methionine | |
| --- | --- | --- | --- | --- | --- | --- |
|  | K_I_ [μM] | Binding energy[kcal/mol] | K_I_ [μM] | Binding energy[kcal/mol] | K_I_ [μM] | Binding energy[kcal/mol] |
| KVRLIQFEKV (68-77) | 3.61 | -7.43 | 0.997 | -8.19 | 0.963 | -8.21 |
| LPALQMFMR (156-164) | 0.081 | -9.67 | 0.103 | -9.53 | 0.093 | -9.59 |
| KKKKYKDKYL (247-256) | 0.067 | -9.78 | 0.059 | -9.86 | 0.085 | -9.64 |
| KHSSIFDQLDVVSYEEVVRLPAFKRKTLV (258-286) | 0.309 | -8.88 | 0.276 | -8.95 | 0.342 | -8.82 |
| KFVYPVPYTTRPPR (309-322) | 0.019 | -10.52 | 0.023 | -10.41 | 0.020 | -10.5 |
| RSQYAHYFDLSLV (423-435) | 0.561 | -8.53 | 0.515 | -8.58 | 0.330 | -8.84 |
